# Smart Healthcare System with Light-Weighted Blockchain System and Deep Learning Techniques

**DOI:** 10.1155/2022/1621258

**Published:** 2022-04-19

**Authors:** Randeep Singh, Bilal Ahmed Mir, Lohith J. J, Dhruva Sreenivasa Chakravarthi, Adel R. Alharbi, Harish Kumar, Simon Karanja Hingaa

**Affiliations:** ^1^Department of Computer Science & Engineering, IEC University, Baddi, Solan, Himachal Pradesh, India; ^2^Department of Mechanical and Intellectual Systems Engineering, University of Toyama, Japan; ^3^Department of Computer Science and Engineering, B M S College of Engineering, Bengaluru, India; ^4^KL Business School, Koneru Lakshmaiah Education Foundation Deemed to be University (KLEF), Vaddeswaram, Guntur District, Andhra Pradesh, India; ^5^College of Computing and Information Technology, University of Tabuk, Tabuk 71491, Saudi Arabia; ^6^Department of Computer Science, College of Computer Science, King Khalid University, Abha, Saudi Arabia; ^7^Department of Electrical and Electronic Engineering, Technical University of Mombasa, Mombasa, Kenya

## Abstract

A radio communication sensor system is a collection of sensor modules that are connected to one another through wireless communication. It is common for them to be battery-powered and responsive to a nearby controller, referred to as the base station. They are capable of doing basic computations and transferring information to the base station in most scenarios. They are also in charge of transporting data from distant nodes, putting a burden on nodes with limited resources, and contributing to the quick depletion of energy in these nodes in the process. Nodes in close proximity to the base station are responsible for more than only detecting and sending data to the base station; they are also responsible for transmitting data from faraway nodes. To reward nodes that perform well, a protocol known as the Improved Fuzzy Inspired Energy Effective Protocol (IFIEEP) employs three separate sorts of nodes in order to provide more energy to those who do not. It takes into account the remaining node energy, the node's proximity to the base station, the node's neighbor concentration, and the node's centrality in a cluster when determining node viability. All of these assumptions are founded on a shaky understanding of the situation. Adaptive clustering must be applied to the most viable nodes in order to identify cluster leaders and transmit data to the base station, in addition to disseminating data across the rest of the network, in order to achieve success. In addition, the research provides proper heterogeneity parameters, which describe, among other things, the number of nodes as well as the starting energy of each node. The percentage gain in-network lifetime when compared to current approaches is minor for smaller numbers of supernodes; however, the percentage gain in the area covered 12.89 percent and 100% when more significant numbers of super nodes are used. These improvements in stability, residual energy, and throughput are accomplished by combining these improvements while also taking into consideration the previously neglected energy-intensive sensing energy aspect. The protocol that has been presented is meant to be used in conjunction with applications that make use of blockchain technology.

## 1. Introduction

In a variety of fields, such as home automation, medical monitoring, military operations (including industrial trolleys), retail and logistics, agriculture (including archaeology, animal husbandry, and flood detection), and others, world-wide-web networks (WSNs) are used in a variety of applications. For the time being, however, WSAN (Wireless Sensor and Actuator Network) is essential for operating requirements. However, it seems that 'actuators' have taken the lead in terms of expressing interest at this moment, rather than WSNs. The network itself should have a genuine interest in the subject matter and should be able to determine what needs to be done next, rather than just receiving information on a topic of interest from a remote location. The system should activate the water sprinkler system instead of only notifying BSC if the temperature of an area climbs beyond a certain threshold, or it should sound an alarm in the fireplace, signaling that there is a fire. To provide an example, if the water heater in the house has been running for four hours, turn it off rather than keeping it running unattended or without somebody home to utilize it or keep it in good working order. Additionally, the wristwatch should sound an alert if the blood pressure (BP) of a patient strolling in the gardens rises beyond the recommended range for that patient. As a result, a family member of the hospital in issue will be notified, allowing for the delivery of critical medical care as soon as reasonably possible.

Each hospital's computerized server now stores information on patients' medical and examination histories, as well as their prescriptions and diagnostic findings, which used to be stored on a paper chart. As a consequence of the use of blockchain technology, data may become more trustworthy, secure, interoperable, and easily accessible. These efforts are being carried out by medical blockchain projects such as MediBlock in Korea and the United States. Using artificial intelligence and blockchain to utilize or exchange medical data in a decentralized manner eliminates the requirement for the patient to serve as an intermediary vendor between the two parties. Patients who had previously sold subscriber data and gave authentic data were denied the right to make a decision and the benefits of trade, even though middle-class vendors benefited financially as a consequence of their acts. The genetic information of subscribers to a medical information service provider is sold to pharmaceutical companies to generate revenue, while the patient community generates revenue by selling data concerning the side effects of anonymous people to pharmaceutical companies. Patients who provided information, on the other hand, had a reduced role in decision-making and received no gain from the sale of their products or services. The integration of artificial intelligence and blockchain, in addition, may reduce the amount of middle-market positions and enable more direct decisions to be made, enabling patients to make better use of their data while simultaneously improving profitability [1–3]. You have the option of deciding how much your data will be sold for and to which company's projects it will be used, and you can also choose to monitor the data's usage after it has been sold. Patient engagement will increase if incentives are supplied, which will benefit both the patient and the data buyer in the long run. The objective is to inspire people to take exceptional care of themselves and to make the most of their good health while they can. Encouraging patients to participate in physical activity, quit smoking, stop drinking, and limit their prescription consumption is a thing that the medical community struggles to do with great success. In the case of medical information systems and healthcare systems that are integrally linked to artificial intelligence and blockchain technology, it is feasible to apply for insurance coverage and to be covered by insurance.

It is necessary to account for data collisions in this model since a large number of communication units are all utilizing a single communication channel at the same time in this scenario.

The task of relaying data from other nodes that are located outside of the network's coverage area, in addition to the regular transmission of information seen by the node, is added to the list of responsibilities. The results of the LEACH [2] simulation have led to the conclusion that the congestion in the area around the BS is caused by a large amount of data being relayed from distant nodes. As a consequence, load balancing is a critical need. Although it is unlikely, it is conceivable that the relaying nodes may fail, creating a vacuum between them and their base station (BS). This will prohibit them from sending data even if they have battery power available. Battery terminology such as “alive” and “dead” refers to a battery that is ready to be used or operational, as opposed to a battery that is unable to be used or operational owing to the depletion of a battery's energy or the physical damage to a battery's terminals. Cluster heads are selected using a stochastic cluster head selection process in LEACH, and as a consequence, asymmetric battery reservoirs are generated in nodes as a result of the technique used to choose the cluster heads. Considering both the remaining energy level of each node and how much energy is required to transmit data is crucial to make an educated choice regarding the cluster head.

Make sure nodes that are doing more activities have enough battery power to last longer, or spread the operating load over a larger number of nodes.

The solution to the issue is represented by the number [3]. First, there is data aggregation or data fusion, which encompasses methods such as data compression, in-network query processing, and other related methodologies, among other things. Because of this, the number of bits broadcasted or received by communication entities is reduced, which has an impact on the amount of energy used directly during the transmission of information. Establishing a variety of distinct networks is part of the second method, which is described below. It is considered to be heterogeneous when a network consists of components that are not similar to one another in terms of structure. Network objects' abilities represent the amount of initial battery power, computation capabilities, and communication range that they have when they first appear on the network. To avoid making any comparisons between the nodes in this research, each one has a different starting battery capacity, but both have the same processing and communication skills. The first strategy, on the other hand, makes use of a network that is normally homogenous in terms of battery capacity, but the second technique does not.

Because a high number of communication units are all using a single communication channel at the same time in this situation, it is vital to account for data collisions in this model.

According to the findings of the LEACH simulation, which was performed, it is expected that a high quantity of data being transferred from distant nodes is a contributing reason to the congestion in the region around the BS. As a result, load balancing is a vital need. The relaying nodes may fail, resulting in a void between them and their base station, even though this is improbable. This will prevent them from transferring data even if they have the battery power to do so on their own. Using battery terms such as “alive” and “dead,” you can distinguish between a battery that is ready to be used or operational and one that has been inoperable as a result of a battery's energy being depleted or the battery's terminals being physically damaged. Cluster heads are chosen using a random number generator in LEACH. It is essential to take into account both the remaining energy level of each node and the amount of energy necessary to transfer data to make an informed decision on the cluster head.

Maintain sufficient battery power in nodes that do a greater number of activities, or distribute the operational load over a wider number of nodes to ensure that nodes that perform fewer activities remain longer.

First and foremost, there is data aggregation or data fusion, which includes technologies such as data compression, in-network query processing, and other related approaches, among other things. In turn, the number of bits transmitted or received by communication entities is lowered as a result, which influences the amount of energy utilized directly during the transmission of data. The second strategy, which is detailed further below, includes the establishment of several unique networks. When a network comprises components that are structurally distinct from one another, the network is said to be heterogeneous. The abilities of network objects describe the amount of initial battery power, computing capabilities, and communication range that they have when they initially emerge on the network, as well as their ability to communicate with other network objects. Each of the nodes in this study has a distinct beginning battery capacity to avoid drawing any comparisons between them; nonetheless, they each have the same processing and communication abilities. When compared to the second approach, the first strategy makes use of a network that is generally homogenous in terms of battery capacity, while the second technique does not.

In the current analysis, an energy-efficient heterogeneous network of nodes that runs at three separate energy levels is taken into account. Even though the gadget's use is out of the usual in terms of its geographical location, the device was designed expressly for this kind of application in mind. This was accomplished as a consequence of the use of fuzzy logic. Many more discoveries have been made, including the mathematically optimal number of particular node kinds for each node, as well the amount of beginning energy that each node should have, among other findings. The findings were also compared to a set of existing protocols, which consisted of a heterogeneous network with two initial energy levels and three inherent energy levels, respectively. Also it is investigated if it is necessary to strike a compromise between network life extension and coverage percent area per unit time to get the best potential results. The currency network protocol is built on the foundation of fuzzy logic, and the results of the simulation show that the protocol is extremely close to real-time or human understanding, resulting in a network that is far more stable and trustworthy throughout its existence. Fuzzy logic is used as the foundation for the currency network protocol. The next section will offer a more in-depth description of the improved fluid inference system in more detail. The improved assumptions, network deployment, clustering, and data transmission mechanisms, to name a few topics covered in detail in this section, as well as the mathematical assessment and computational interpretation of optimal heterogeneity parameters, are just a few examples of the topics covered in this section.

## 2. Related Work

The related work in the domain has been summarized in Table 1.

### 2.1. Literature Gap

- Fuzzy factors, such as waste energy, proximity to the base station, concentration, and center, are all examples of variables that may be employed
- Additional improvement in adaptive cluster head selection is possible, as is the case in the previous section [7]
- To establish the best heterogeneity parameters, a model must be developed

#### 2.1.1. Fuzzy Inference System

The characteristics of human thinking, on the other hand, are difficult to translate into binary computer logic, which can only be expressed as yes or no. When it comes to fuzzy sets, fuzzy logic incorporates dialectical factors into human thinking by introducing them into the set of possibilities.

**Figure 1:**
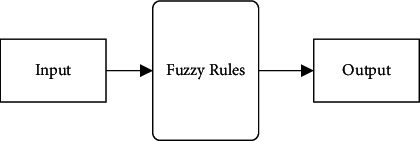
Input fuzzy system.

Following the computation, as seen in Figure 1, the fuzzy inference system (FIS) provides crisp output values as a consequence of the process. Because of the existence of a knowledge base, the input values change, and these fluctuating inputs are sent to an inference mechanism, which applies a variety of fuzzy if-then rules to construct conclusions about the outside world. The output generated is then aggregated and defuzed with the help of the knowledge base, resulting in a set of potential output values to select from. The Mamdani type of FIS was used in this work, which means that the output of each if-then rule is a fluctuating collection of values rather than a single value.

### 2.2. Fuzzy Improved Model (Improved Model Fuzzy)

A network node may only be assigned to a cluster member if the CH deems that it has sufficient energy resources to acquire, aggregate, and send database data from its cluster members, regardless of where the cluster member is located in the network hierarchy. Thus, while selecting a CH, it is critical to include the residual node energy as one of the key metrics to consider. LEACH [8] and similar direct transmission protocols do not evaluate the node's remaining energy before allocating the function of CH, resulting in early battery energy depletion for nodes close to the base station or frequent allocation of the function of CH for nodes farther away from the base station. LEACH [8] and similar direct transmission protocols do not evaluate the node's remaining energy before allocating the function of CH. LEACH and other direct transmission protocols do not take into account the amount of energy that a node has left when assigning the function of CH.

The radio energy model shows that when a knot is near a base station, it is often supplied with CH, as represented on the diagram. However, such nodes should not be overloaded again since doing so might lead them to expire prematurely. This is done to lower the overall amount of energy used by the network, which is particularly important in a network with limited available energy. This might then result in a data hole, which is defined as not transmitting any data to the base station even though distant nodes are still alive and well. The following is the distance between nodes and the base station: As soon as the transmission radius of a node is determined, the concentration of neighbors within that radius may be calculated. Distance between nodes and base station: This indicator represents the degree to which data redundancy occurs in the event of a high node density in a particular geographic location. With increasing concentration, the chance that nodes will be designated as CHs to avoid data duplication increases (central hubs).

The distance between a node and the node's origin is defined as the centrality of the node (the center of the cluster in which it is situated). The sum of the square distances between all of the other nodes and the single node under examination are calculated by the base station, which is located at the center of the network. Additionally, it is hypothesized that the lower the center value of a node, the less the energy used by subsequent protuberances in transiting their data via that node to its eventual destination. It is vital to underline once again that nodes such as these should not be overburdened, and that duties should be assigned to all nodes at the same time to establish an equitable energy distribution throughout the network, as has been done before.

The different components of the fuzzy inference system that was used in the present investigation are listed in the following sections.

**Figure 2:**
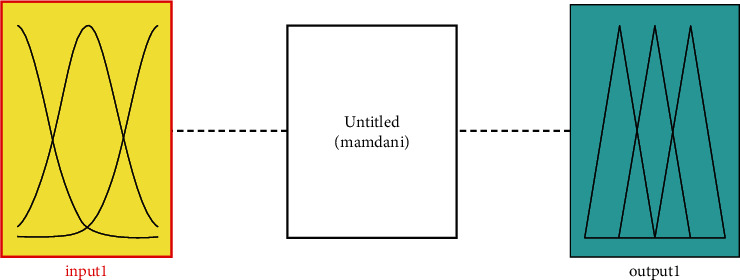
Improved : System model.

Using a single membership function, fuzzification is the process by which the input values are transformed into dialect variables. On the other hand, as shown in Figure 2, certain membership tasks are carried out to varying degrees.

**Figure 3:**
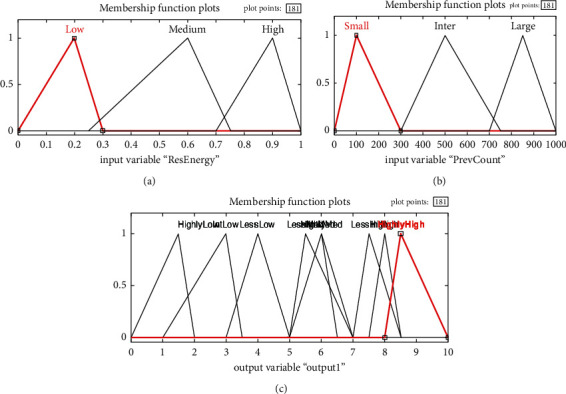
Description of input membership functions. (a) Node's residual energy. (b) Station input. (c) Concentration of nodes.

It is possible to find 180 if-then rules in the knowledge base or database, which contains the specification as well as the definition for if-then rules. To make it simpler to grasp the various regulations, they have been shown as surface plots to make them more visible (Figure 3).

It does this by using membership functions to translate the output uncertain productions from the implication device into crisp standards [8]. When the centroid of area approach is used, defuzzification may be achieved more effectively.

**Figure 4:**
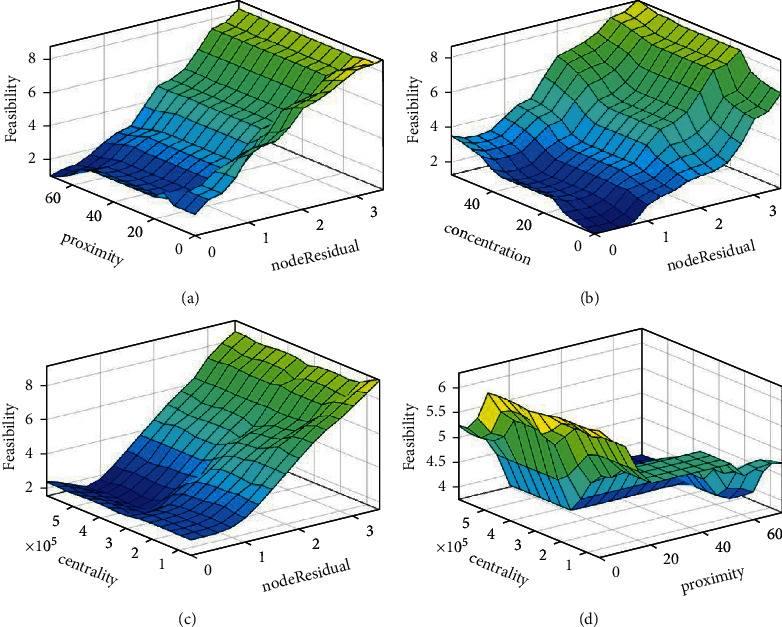
Surface plots representing rule base. (a) Proximity vs. node residual energy. (b) Concentration vs. node residual energy. (c) Centrality vs. node residual energy. (d) Centrality vs. proximity to the base n.

Figure 4 represents the surface plots representing rule base.

## 3. Proposed Energy Protocol with Blockchain

The extra ledger data system, DLT (Distributed Ledger Technology), consists of validating machines with Ethereum. It can execute operations that are equivalent to those carried out by the Bitcoin cryptocurrency. The Ethereum Virtual Machine (EVM) allows for the building of immutable computer logic that can be constructed on top of the Ethereum blockchain and then executed in public books. When it comes to the introduction of smart contracts and the execution of smart contracts, both need the accumulation of power by Ethereum, which is accomplished by the expenditure of a certain amount of money known as the Ethereum cryptocurrency (which is used to do this task) (also known as the Ethereum token). The following section describes the fundamental steps involved in executing a smart contract after it has been registered in the ledger. It is meant for smart contracts to operate with specific input data provided by transactions. Transaction data is the term used to describe this kind of information. It is possible to guarantee that money movement during transactions and changes in the state of EVMs are completely transparent and can be inspected by any party involved in the transaction [4, 5]. The upshot is that instead of using an old-fashioned squashing function scientists and engineers throughout history have employed the sigmoid function to solve difficulties, as well as the tahn function, which is a soft step function that may be identified, to accomplish their objectives. We must emphasize that although the sigmoid function and the step function are quite similar in appearance and function, there are significant differences when compared to the biological brain, which cannot work properly unless there is some passage of time. While the input value for artificial neural networks may be supplied regardless of the time flow, the output value for artificial neural networks has been predetermined (ANNs). In contrast, researchers previously believed that artificial neural networks, which are distinct from the biological brain, would have difficulty calculating as fast computers because of their high computing performance, and that the artificial neural network model was on the verge of becoming obsolete as a result. As a result of its structural organization, which is modeled on that of the human brain, this artificial neural network serves as the foundation for artificial intelligence technologies.

### 3.1. Suppositions

**Figure 5:**
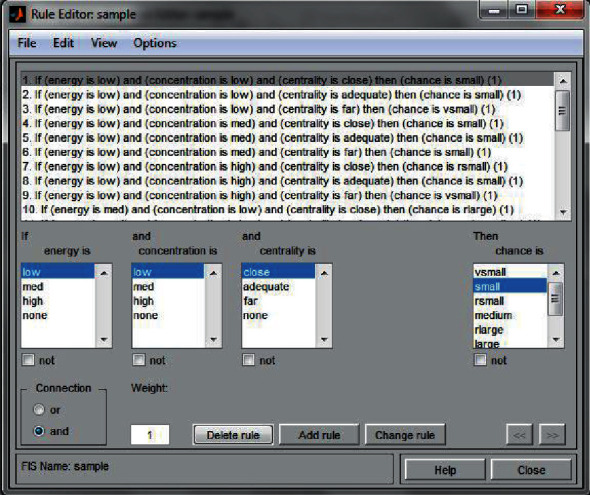
Description of output membership function.

**Figure 6:**
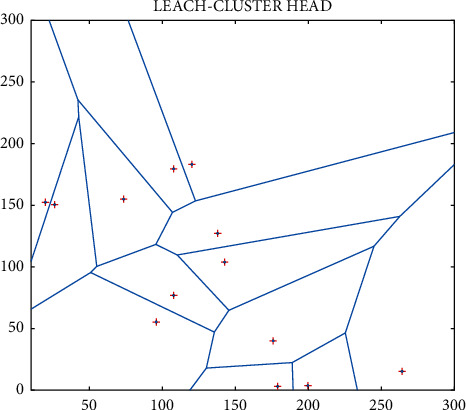
Improved : Network architecture.

1. Network sensor nodes have comparable capabilities, are stationary, and are permanently added
2. Network sensor nodes have comparable capabilities, are stationary, and are permanently data-bound
3. As a result of the exclusion of propagation lag, the delay is defined as the time it takes for data to propagate from one node to the next or for a round of propagation to complete
4. The communication channel is symmetric for every signal-to-noise ratio that is used in the experiment
5. Fourth, the Time Division Several Access (TDMA) protocols have been established to allow multiple users to access shared channel resources at the same time [9]

- Figure 5 represents the membership function. The first-order radio energy model has been applied to the problem of energy dissipation among communication entities [10]. Energy consumption by nodes varies in proportion to the distance between them or the role that has been allocated to them [11], which may include tasks such as data originator, relay node, or cluster leader, among other things.
- Similar to this book, each node starts with the same initial energy model, which is the same as in this book.
- The fact that nodes are aware of their relative position about other nodes based on the received sign strength indication does not imply that they are capable of giving [12] precise location information.
- The broadcast range of each node may be modified depending on the circumstance. As an example, if no other network node can transfer the data to the backend server, then a node may be able to communicate the data directly to the backend server.
- Implementing heterogeneous networks: This section addresses the process of putting heterogeneous networks into operation.
- In the terrain is a subsection known as the “inner circle,” which has a radius one-hundredth the length [13] of the field's side and a radius one-hundredth the length [14] of the field's side, respectively. Nodes that are responsible for relaying a huge quantity of data are separated by a distance that is comparable to a single-hop distance, resulting in the consumption of a tremendous amount of energy between the nodes. To deploy the various kinds of nodes, a homogenous spatial poison process [15] with the following parameters has been employed: Standard nodes that are dispersed across the network are called as shown in Figure 6.

Essentially, the idea behind the aforementioned deployment is to provide more energy to nodes that are near the base station (supernodes), allowing them to work for a longer period [14] because they have more information to broadcast than they would otherwise be able to [16] (in the role of relay nodes). If such nodes fail, a data void occurs, which means that either far-flung nodes are unable to transmit data as a result of the failure of intermittent forwarding nodes, or they consume more energy than is necessary as a result of the failure of relay nodes as a result of the increase in distance between the nodes in question, resulting in data void. More recently [17], advanced nodes beyond the inner circle have been deployed to supply additional energy to regular nodes, while normal nodes, although being less common in LEACH [18], have been applied in the context of heterogeneous networks [19].

Alternatively, the examination of nodes for feasibility is achieved by the use of fuzzy logic, and this research is the first step in determining the clustering process among the nodes [18]. Next, the feasibility values of the nodes that are eligible to be CH are sorted in decreasing order, and the nodes with the lowest ten percent of feasibility values are eliminated from the process of CH selection, resulting in an initial set *G* of nodes that are eligible to be CH for the current epoch [16]. To serve as a central hub (or CH) for the game's central hub (or CH) in each round, the top 5 [19] percent of all possible nodes are picked for each round. Initial selections for CH nodes are made ineligible for selection as CH for the current epoch early on, and this process continues until all of the eligible nodes from *G* have been selected as CH for the first time [20]. Indefinitely, until all nodes have been removed from the system, this process of selecting cluster chiefs may be repeated.

### 3.2. The Dissemination of Information

For data to be effective, it must be moved from a lower level in the hierarchy to a higher level in the hierarchy or the base station. This hierarchy includes the sensor node, the cluster head, and the next cluster head, among other things. The nodes in the lower and higher levels of this hierarchy are coming closer to the base station as the hierarchy progresses.

#### 3.2.1. The Heterogeneity Parameters That Produce Effective Results Best

If we want to understand how to analyze a heterogeneous network, we must first figure out how to determine the optimum number of certain types of nodes or the best amount of energy in a particular kind of node [16]. The word “heterogeneity” refers to the differences in the amount of initial energy that each of the nodes possesses at the commencement of the simulation. As a consequence, it is possible to identify the optimal ratio between the other two variables in a simulation.

#### 3.2.2. Character of Utility

This study's nature of protocol's usefulness was developed using the framework outlined in the next section, which may be summarized as follows: 1. The system timeline is defined as the period that has elapsed between the first placement of the network and the demise of the final protuberance in the network [16]. 2. Presently, there are nodes providing service to the network, and this number reflects the number of nodes that are currently available for use by clients.

The phrase “number of dead nodes per unit time,” in this context, refers to *b*. the prompt largeness [21] of the count of protuberances that have depleted their battery resources and are thus no longer capable of communication with the base station.

The amount of residual energy (measured in battery volume) obtainable in the system per unit of time may be tracked using this account, and the account is updated in real-time when the network's residual energy changes [22]. This is the total quantity of energy created by all nodes, regardless of the kind of node that is being utilized to generate the energy.

When calculating the amount of energy used by the execution of a specific strategy during the round under consideration, the amount of energy consumed by the execution of a specific strategy during the round under consideration is taken into account [23]. Among other things, this energy is required for the operations of detecting, collecting, calculating, and transferring data from nodes to the base station.

Honghai Zhang et al. [24] indicate that at least twice the length of the sensing range should be available for radio transmission for a collection of operational nodes to stay linked.

The percentage of area covered about the length of the network is an important consideration to keep in mind when making this comparison [25]. When it comes to network longevity, the amount of time that has passed since the network was first deployed and the amount of time that has passed after the network was deactivated and decommissioned are both important considerations. However, although it is typical to speak about extending the length [26] of a network by making optimal use of existing resources, it is equally important to consider the amount of territory covered by the nodes throughout the planning process [27].

#### 3.2.3. Outcomes

Several hours of debate resulted in the decision that the improved would run on *m* = 0.7, which means that around 70% of the nodes got some more energy as compared to the initial Eo value. To be more specific, this was done to evaluate how the increased amount of energy that was provided to the nodes influenced their performance [28]. Given that the network has a constant value and that the total quantity of energy in it is constant, we can use it to evaluate the value of utilizing the network by varying the value of mo between the ranges of 0.2 and 0.90.

Using a mo value equal to 20% of the total energy nodes, it is possible to obtain the longest stable lifetime, which means that there are almost no dead or dying nodes over an average of 1000 to 7000 nodes as provided by the most sophisticated nodes (see Figure 1.7a). Whatever way you look at it, there is no doubt that the steepness that occurred before was caused by the death of ordinary nodes [29], which was then followed by the death of advanced and supernodes [30]. The number of supernodes is much greater when comparing mo = 0.9 to other mo values (such as mo = 0.1 and 0.6), and these supernodes contribute to a higher number of live nodes than when comparing other mo values (such as mo = 0.1 and 0.6).

**Figure 7:**
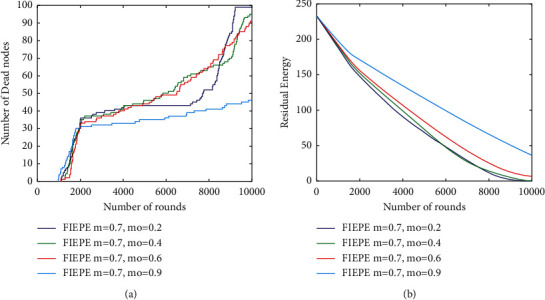
The behavior of improved changes in response to the number of supernodes. (a) Overall dead nodes; (b) network residual energy.

Figure 7 represents the performance metrics. Even though the amount of dead nodes, the amount of residual vigor, and the amount of energy expended in each round all seem to be advantageous for a greater number of supernodes in this scenario [31], the proportion of area covered per unit of time in this scenario is much lower. As an explanation, the supernodes have been put in an inner circle with a radius that is about equal to the distance between two nodes in a single hop, which is why this is occurring. When this occurs, the covered zones within a certain radius begin to overlap, resulting in a decrease of the percentage of the overall area covered.

In the correlative investigation, a total of three heterogeneous wireless sensor network protocols were employed, including SEP [32] for two levels of heterogeneity, three-level SEP for three levels of heterogeneity, and a hybrid protocol for three levels of heterogeneity. Several characteristics of these protocols were shared with the SEP protocol, including the fact that they started with the same amount of initial energy as the network and had the same number of nodes as the network [31].

Initial calculations were made to assess how much of a difference there was between the SEPs of two distinct levels of nodes, namely, normal and advanced. In the initial investigation, which was carried out in two parts, the SEP for two different types of nodes was determined and analyzed. Based on the above results, it appears that the number of advanced nodes can be varied between 0.22 and 0.90 when the value of the parameter *m* = 0.22 to 0.90 is changed.

Depending on whether you have a certain quantity of total energy that is equal to or more than the amount of energy delivered by the source of energy, the efficiency of improved model may vary from 20 to 90 percent. In this study, the amount of additional energy [33] required by 20% of advanced nodes is significant, and as a result, all normal nodes die after a certain period of time, whereas progressive nodes remain alive for a longer period of time due to the fact that data from a smaller number of nodes is being gathered. The possibility of seeing a similar pattern for varying percentages of advanced nodes [31] is also possible. The fact that the network's chronology stays constant until the death of the first node, regardless of the number of progressive nodes that are present in the network at any one point, is also worth mentioning.

**Figure 8:**
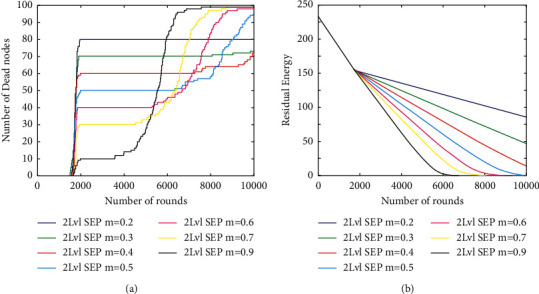
Overall performance metrics. (a) Total dead nodes. (b) Total residual energy.

According to Figure 8(b), the complete remaining potency of the system per unit of time stays constant up to and including the death of the first node [33], regardless of the state of the other nodes in the network [34, 35]. As a consequence, the procedure is performed over and over again until the simulation period has been reached.

**Figure 9:**
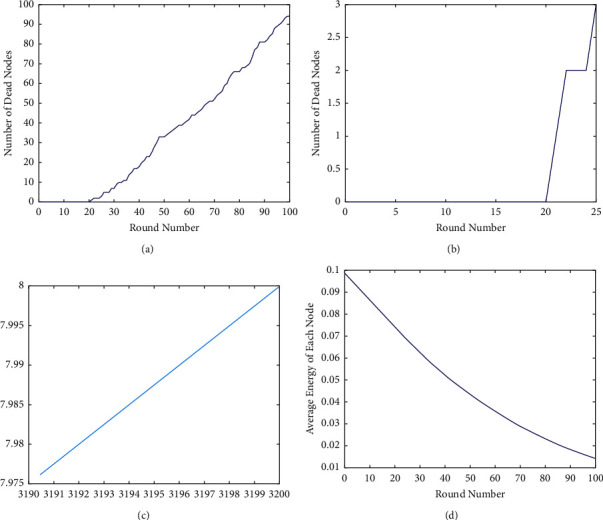
Proposed methodology performance metrics. (a) Dead nodes. (b) Residual energy. (c) Total energy. (d) Overall area.

The SEP for three levels has also been taken into consideration, which means that three types of nodes (normal, advanced, and supernodes) have all been studied, with the number of initial energies increasing from the first level to the final. This results in an increase in the number of supernodes (mo) for a given amount of total energy equal to the improved, from 0.1 to 0.9. However, a preset percentage of additional energy nodes is kept to allow comparison with present protocols at a later period (*m*). Following that, DEEC has been investigated for three unique types of nodes and situations that are identical to those mentioned above in the three-level SEP framework, and the findings have been made available for review. On the right-hand side of Figure 9(a), you can see a bar graph indicating the number of dead nodes per unit time, with each node representing a unit of time in the graph [35]. Furthermore, it has been shown that the death pattern of nodes for three-level SEP has no influence on the number of superprotuberances that are used; however DEEC is quite sensitive to variations in the number of amazing protuberances that are used. According to [12], when looking at the system's overall lifetime, the DEEC system has a flat saturated region, with contributions from advanced nodes growing in proportion to the number of supernodes present in the system as the system develops. It appears that the amount of total residual energy in the network is a function of the number of wonderful bulges in the system, which is depicted in Figure 9(b); however, as the number of superbulges in the network increases, it appears that the amount of residual energy is also increasing in the network, as depicted in Figure 9(c). Following that, Figure 9(d) depicts the quantity of area that is covered in a certain period of time. However, increasing the number of beautiful protuberances enables the DEEC to exhibit a bigger amount of portion enclosed for a longer length of time, which is consistent with the prior results [36].

**Figure 10:**
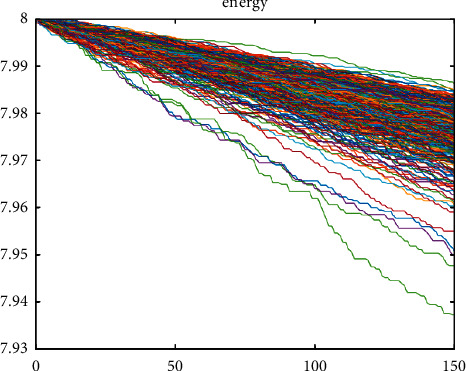
Assessment of improved model with 2 levels.

At the conclusion, we will give a comparison of improved and SEP at the 2, 3, and DEEC stages.  Figure 10 represents the energy at various levels of protocol implementation.

This is shown in Figure 10, which shows that the remaining liveliness in this situation is adequate until the death of the final node [37].

Table 2 summarizes the percentage gain in improved protocol, the fuzzy stimulated liveliness well-organized protocol for diverse wireless sensor networks [38], as compared to the other protocols under consideration in terms of the number of nodes dead for the two parameters considered to be *m* = 0.7 and mo = 0.2. The number of nodes that die during the entire computation time is only 16 percent; as a result, the maximum amount of residual energy can be observed; however, the percentage of area covered by this scenario is only 13.29 percent, despite the fact that 51 percent of nodes are still alive during the entire computation time [39]. This demonstrates that when the number of supernodes changes, there is a tradeoff between the number of nodes that are dead and the percentage of area covered by the supernode network [40].

## 4. Conclusion

We have created a protocol for a more diverse wireless device system that does not allocate cluster heads to nodes based on a probabilistic threshold, regardless of node load, residual energy volume, or data redundancy, but instead calculates cluster heads based on residual energy volume [41]. Additionally, the nodes with more significant responsibilities are supplied with greater energy, allowing them to do their tasks. Following that, the optimal heterogeneity characteristics that had been derived theoretically were used to predict the proper amount of additional energy that was required for a certain node type that had been found via experimentation [42]. According to the results of a simulation of improved protocol with SEP at two energy levels, three energy levels, and DEEC, improved protocol [43] is more lucrative than SEP. The same holds true when considering a larger proportion of the region covered by a comparable number of leftover energies and energy intake of each cycle.

## Figures and Tables

**Table 1:** Improved: Related work.

Reference	Advantages	Remarks
LEACH 2002 [4] is used as an example.	Among the earliest protocols for data transfer in a WSN it is simple. Because of threshold-based stochastic cluster head selection, there is a bottleneck in the area surrounding BS. In the case of a heterogeneous network, this rule does not apply.	The solution was to create heterogeneity among nodes by assigning them increasingly difficult tasks to complete.
Seema Bandhopadhyay and colleagues 2003 [5]	It was suggested that raising the degrees of hierarchy would result in greater energy savings. Communication is only possible via hierarchies in lag.	When selecting a CH, it is necessary to take into account the residual energy of the node.
Liang Ying and colleagues [6] published their findings in 2005.	LEACH's stochastic cluster head selection has been optimized.	When selecting a CH, it is necessary to take into account the residual energy of the node.

**Table 2:** Percentage gain in improved protocol, the fuzzy inspired energy-efficient protocol for heterogeneous wireless sensor network as compared to existing protocols.

Number of nodes dead	2-level SEP	3-level SEP	3-level DEEC
1% nodes dead	−11.19%	−10.07%	−50.38%
50% nodes dead	28.11%	23.89%	6.96%
80% nodes dead	27.17%	16.83%	10.32%
100% nodes dead	6.60%	1.60%	5.29%

**Percentage area covered**			

1% nodes dead	5.58%	1.96%	5.19%
50% nodes dead	11.97%	1.22%	1.68%
80% nodes dead	26.66%	8.86%	9.72%
100% nodes dead	100%	12.89%	100%

## Data Availability

The data that support the findings of this study are available on request from the corresponding author.
